# Effect of Solid-State Fermented Wheat Bran Supplemented with Agrimony Extract on Growth Performance, Fatty Acid Profile, and Meat Quality of Broiler Chickens

**DOI:** 10.3390/ani10060942

**Published:** 2020-05-29

**Authors:** Boris Semjon, Martin Bartkovský, Dana Marcinčáková, Tatiana Klempová, Lukáš Bujňák, Marek Hudák, Iveta Jaďuttová, Milan Čertík, Slavomír Marcinčák

**Affiliations:** 1 Department of Food Hygiene and Technology, University of Veterinary Medicine and Pharmacy in Košice, Komenského 73, 041 81 Košice, Slovakia; boris.semjon@uvlf.sk (B.S.); martin.bartkovsky@uvlf.sk (M.B.); iveta.jaduttova@student.uvlf.sk (I.J.); 2 Department of Pharmacology and Toxicology, University of Veterinary Medicine and Pharmacy in Košice, Komenského 73, 041 81 Košice, Slovakia; dana.marcincakova@uvlf.sk; 3 Institute of Biotechnology, Faculty of Chemical and Food Technology, Slovak University of Technology, Radlinského 9, 812 37 Bratislava, Slovakia; tatiana.klempova@stuba.sk (T.K.); milan.certik@stuba.sk (M.Č.); 4 Department of Nutrition, Dietetics and Animal Breeding University of Veterinary Medicine and Pharmacy in Košice, Komenského 73, 041 81 Košice, Slovakia; lukas.bujnak@uvlf.sk (L.B.); marek.hudak@student.uvlf.sk (M.H.)

**Keywords:** nutrition, broiler, meat quality, gamma-linolenic acid, agrimony extract

## Abstract

**Simple Summary:**

The current work evaluates the application of solid-state fermented wheat bran supplemented with agrimony extract in broiler nutrition. Broiler production parameters, blood and bone variables and meat quality were analysed. The quality of breast and thigh meat was evaluated by the use of physicochemical variables, fatty acid profile, lipid oxidation and sensory variables. The presented results showed that supplementation of the broiler diet with fermented feed positively influenced the quality of the produced breast and thigh meat. The application of fermented feed increased the nutritional value of broiler chicken meat, as shown via the positive modification of the fatty acid profile, without affecting sensory quality.

**Abstract:**

The impact of the broiler diet modification on the following parameters was evaluated: meat quality, carcass traits, and bone and blood parameters. One hundred twenty one-day-old COBB 500 broiler chickens were assigned to three experimental groups (40 birds per group) with four replications (10 per pen) for 35 days of fattening. The control (C) was fed a basic feed mixture. The diet supplemented with 10% of fermented feed (FF10) and additionally supported by 0.2% of agrimony extract (FF10 + AE) was applied to the second and third groups, respectively. FF10 showed both a lower average daily feed intake and total feed consumption when compared to that of C (*p* < 0.05). Lower concentration of alkaline-phosphatase and calcium and higher total lipids and triglycerides in blood were observed in FF10 + AE. Breast and thigh meat showed a lower content of polyunsaturated fatty acid n-3 and n-6 in the FF10 + AE group (*p* < 0.01). The increase of gamma-linolenic acid content in breast and thigh meat samples obtained from the experimental groups was significant (*p* < 0.001 and *p* < 0.05; respectively). Lower lipid oxidation was observed in the thigh muscle of the FF10 + AE group on the first day of storage (*p* < 0.01). The current study indicates that FF10 + AE supplementation can be successfully applied to enhance broiler performance and meat quality.

## 1. Introduction

Successful broiler chicken meat production depends on feed as one of the main contributory factors. Parameters that affect meat quality are complex and occur throughout the production chain [1]. Another contributory factor could also be the lack of another type of meat in certain locations afflicted by disease, e.g., in regions where African swine fever occurs in pigs. Development of newer, more efficacious techniques to enhance the health, and in turn, the production of poultry is vital for the modern, ever-evolving broiler chicken meat industry [2]. Due to a global rise in the price of feed ingredients, there is now an increasing trend for the modern poultry industry to use agro-industrial residues as feed ingredients [3]. Agricultural byproducts are inexpensive, but largely useless since their abundant fibre content limits their use as feed for monogastric animals [4]. However, these byproducts can be converted into value-added renewable products and also animal feeds by using the process of fermentation [5].

Solid-state fermentation (SSF) is the oldest known fermentation processing technique. It has been employed to enhance nutrient bioavailability, inhibit gut pathogenic bacteria and reduce antinutritional factors in plant protein sources, resulting in improved nutrient digestibility, and thereby improving the performance and gut health of broiler chickens. The SSF process involves growing microorganisms on solid materials under controlled conditions in the absence of free water [6]. The main important objective of SSF is the production of enzymes, organic acids and other metabolites during microbial growth [7]. After fermentation, the solid-state fermented feed is enriched with various types of metabolites, according to the employed microbe [8]. There are various types of microorganisms (such as bacteria, yeast, filamentous fungi) that can be applied in the fermentation process [9]. In recent years, several studies have been published, which detail the use of filamentous fungi in SSF and its successful application in broiler nutrition. Specific examples include the use of *Umbelopsis isabellina* CCF2412 on cornmeal [7,10,11], *Trichoderma pseudokoningii* on wheat bran [4], *Acremonium charticola* and *Rhizopus oryzae* on cassava pulp [3], *Cunninghamella elegans* CCF 2591 on spelt brans [12], *Trichoderma viride* on copra meal [13], and *Trichoderma virens* on palm-fruit husk [14]. Čertík et al. [15] observed that SSF with *Cunninghamella echinulata* ATHUM 4411 produces mainly gamma-linolenic acid (18:3, GLA). GLA is an isomer of *α*-linolenic acid, and it is classified as an *ω*-6 fatty acid [15].

Chicken meat is currently the most preferred meat by consumers, owing to its low cost, ease of culinary preparation, and its nutritional characteristics [7]. The relevance of broiler chicken meat for humans has been evaluated by the Food and Agricultural Organization (FAO) which, in a recent document, stated that it contains high-quality protein and a low level of fat, but with a desirable fatty acid profile [16], which could help in the overall prevention of cardiovascular diseases in the human population [17,18,19]. To increase the proportion of polyunsaturated fatty acid (PUFA) in the tissues of monogastric animals, feeds with a high content of PUFA, e.g., vegetable oils, oilseed seeds, or fish oil, are commonly used [20]. However, by fermentation of cereal byproducts with nontoxinogenic filamentous fungi strains, it is possible to produce PUFA-enriched feed [15]. The GLA materials that are produced after SSF processing could be used directly as feed additives (without extraction of GLA oils) to modify the fatty acid profile in poultry [21]. Broiler chicken nutrition enriched with GLA could result in an increased proportion and adjusted ratio of *ω*-3 and *ω*-6 fatty acids in broiler meat. The disadvantage of the higher PUFA content in the meat of broiler chicken lies in the fact that it is rather susceptible to the oxidation process. To find the best method to prevent PUFA oxidation, several studies in recent years have been conducted. Supuka et al. [22] observed that supplementation of broiler chicken feed with plant additives, such as agrimony extract, beneficially influenced the oxidative stability of thigh meat and thus improved meat quality.

This experiment was primarily designed to examine the effect of 10% addition of wheat bran after SSF by *Cunninghamella echinulata* ATHUM 4411 in the diet of broilers, and a supplementation addition of SSF wheat bran with agrimony extract (*Agrimonia eupatoria* L.) on broiler production parameters and the quality of the produced breast and thigh meat. In particular, this study evaluates the impact of the fermented feed and agrimony on broiler chicken production variables, biochemical blood and bone parameters, and fatty acid profiles of broiler chicken meat. The current study indicates that supplementation of fermented feed in broiler nutrition could be used to enhance the performance and meat quality, especially in fatty acid profiles.

## 2. Materials and Methods

The animal protocol for this research was approved by the Ethical Committee for Animal Care and Use of the University of Veterinary Medicine and Pharmacy in Košice (Košice, Slovakia). The experiment was carried out in accordance with the “European Directive on the protection of vertebrate animals used for experimental and other scientific purposes” [23] and with the consent of the State Veterinary and Food Administration of the Slovak Republic no. 12492/10-221 on the premises of the Clinic for birds and exotic animals at the University of Veterinary Medicine and Pharmacy in Košice (Košice, Slovakia).

### 2.1. Preparation of Fermented Feed

Fermented feed (FF) was prepared by fungal solid-state fermentation, according to Čertík et al. [24]. The strain *Cunninghamella echinulata* ATHUM 4411 and wheat brans as a substrate were used for the preparation of the FF product. Wheat brans were received from Biomila ltd. (Šajdíkovce Humence, Slovakia). *Cunninghamella echinulata* ATHUM 4411 was obtained from the Culture Collection of Fungi, Charles University, Prague, Czech Republic. The culture was maintained on potato–dextrose agar at 4 °C and reinoculated every three months. The spore suspension for inoculation of SSF was prepared from a seven-day-old mycelium grown on rice with sterilised distilled water to a final concentration of 1–2 × 10^6^ spores/mL. Autoclavable microporous high-density polyethylene (HDPE) bags were filled with 100 g of wheat bran, moistened by the addition of 100 mL of distilled water for 2 h at laboratory temperature and subsequently autoclaved (105 kPa, 105 °C, 1 h). The cooled substrate was inoculated with 20 mL of the spore suspension (1–2 × 10^6^ spores/mL). The HDPE bags were closed, and the substrate was incubated at 25 ± 1 °C for 5 days. The fermented substrate was then oven-dried at 65 °C to constant weight. The final FF product after SSF processing contained 2.1 ± 0.4 mg/g GLA.

### 2.2. Birds, Housing, Diets, and Experimental Design

For the trial, 120 one-day-old COBB 500 (*Gallus gallus domesticus*) male broiler chickens were purchased from a commercial supplier (Hydina Slovensko ltd., Lieskovec, Slovakia). All the birds were vaccinated by the birds’ supplier at hatching against Newcastle disease and infectious bronchitis. The broilers were randomly divided into one control (C) and two experimental groups, each group consisting of 40 chickens, with 4 replications (10 per pen). Chickens of the first experimental group were fed a diet enriched with supplementation of 10% FF (FF10) and those of the second experimental group were fed a diet enriched with 10% supplementation of FF supported with the addition of 0.2% agrimony extract (AE; *Agrimonia eupatoria* L.) into drinking water (FF10 + AE). FF and AE were provided after the 10th day of fattening to broilers belonging to both experimental groups. In the process of feeding and, thus, fattening broilers, three phases were used: a starter diet during the first 10 days of fattening, a growing diet from days 11 to 28 and the final diet from days 29 to 35. The control group of animals was fed with basic feed mixtures (starter, grower, and finisher) without feed mixture supplementation of FF or the addition of AE in the water. The main components of feed mixtures were wheat, corn, soybean meal, rapeseed cake and sunflower meal. The composition of FF and basic feed mixtures are presented in Table 1. Broilers were reared on deep litter under controlled conditions. During the whole time of fattening, the light and temperature regime was monitored [25]. During the trial, a 24-h light regime was set on the first day, and this was subsequently decreased to 18-h. The ambient temperature was gradually lowered from an initial level of 33 °C (day 1) to 21 °C (day 24), and the ambient humidity was maintained at approximately 70%. The animals had access to water and feed ad libitum during fattening. Clinical symptoms of disease and abnormal mortality were not observed during the fattening period. Mortality was recorded only for two members from the control and FF10 experimental groups (one bird from each group), but it was not related to the broilers’ diet modifications. The body weight of individual broiler chicks was measured at weekly intervals, feed consumption was recorded each day, and the feed conversion ratio was calculated at the end of the experiment. The carcass yield was determined as a proportion of the body weight before slaughter and after evisceration.

### 2.3. Feed Samples Collection and Analysis

The dried fermented wheat bran (fermented feed—FF) was supplemented as a feed ingredient with a 10% concentration in the experimental diets. The composition and nutrient content is shown in Table 1. The FF (10%) was well-mixed with other ingredients of feed before being fed to the chickens. The characteristics of the applied fermented product were the following: dry matter 96.3%, crude protein 203.7 g·kg^−1^, crude fat 55.5 g·kg^−1^, crude fibre 119.1 g·kg^−1^, starch 159.2 g·kg^−1^, calcium 1.66 g·kg^−1^, and phosphorus 8.10 g·kg^−1^ of dry matter (DM).

The same basic ingredients for the control and experimental groups were used in the study. Feed and water were allowed on an ad libitum basis.

Each diet had similar metabolisable energy (ME) and crude protein (CP) content. Diets were formulated according to the recommended nutrient content for poultry [26]. The chemical compositions of fermented feed and diets were determined for dry matter, crude protein, crude fat, crude fibre, starch, and total phosphorus according to the EC Commission Regulation 152/2009 [27]. The metabolisable energy value of diets was calculated with the formula according to the EC Commission Regulation [27].

The DM of the diets was determined by drying at 105 °C and weighing. The nitrogen content was determined using the Kjeldahl method via a Kjeltec 2300 Auto analyser (Foss Tecator AB, Höganas, Sweden), and the CP (crude protein) contents were calculated by multiplying the nitrogen value by a coefficient of 6.25. The Soxhlet method was used for the determination of total fat. The fat was extracted in a 2-unit extractor (Det Gras J.P. Selecta S.A., Barcelona, Spain), using petroleum ether. Crude fibre was determined by defatting the well-dried samples, separating the residue in a fibre extractor (Dosi-Fibre extractor, J.P. Selecta S.A., Barcelona, Spain). The starch content of diets was determined polarimetrically using an automatic polarimeter (AP—300, Atago, Japan). The feed samples were analysed for the presence of Ca (calcium) using the flame method of an atomic absorption spectrometer (Unicam Solar 939, Camberley, Surrey, UK). The determination of total dietary phosphorus was performed using the photometric method.

Fatty acid profiles of FF and feed mixtures were determined by measurement of the produced methyl esters by gas chromatography according to Čertík et al. [24]. The gas chromatograph (GC-6890 N, Agilent Technologies, Santa Clara, CA, USA) was equipped with a capillary column DB-23 (60 m × 0.25 mm, film thickness 0.25 μm, Agilent Technologies, Santa Clara, CA, USA) and an FID detector (constant flow, hydrogen 40 mL/min, air 400 mL/min, 250 °C). The analysis was performed under temperature gradient (130 °C for 1 min; 130–170 °C at 6.5 °C/min; 170–215 °C at 2.7 °C/min; 215 °C for 7 min; 220–240 °C at 2 °C/min; 240 °C for 2 min) with hydrogen as carrier gas (flow 2.1 mL/min, velocity 49 cm/s, pressure 174 kPa) and a split ratio of 1/20 (inlets: heater 230 °C, total hydrogen flow 114 mL/min, pressure 174 kPa).

The fatty acid methyl ester peaks were identified by authentic standards for a C4-C24 fatty acid methyl ester mixture (Supelco, Bellefonte, PA, USA) and quantified by an internal standard of heptadecanoic acid (C17:0, Supelco, Bellefonte, PA, USA). The fatty acid concentration was evaluated with ChemStation software B0103 (Agilent Technologies, Santa Clara, CA, USA). All the values were the results of triplicate determination.

### 2.4. Collection of Samples From Broilers

On day 36 of the trial, 120 broiler chickens from four replicates (10 birds per pen), after a 12-h hunger strike, were individually weighed, euthanized by cervical dislocation and were then immediately bled. After slaughtering and the removal of the head, hock cut and evisceration, the carcass weight was recorded. The carcass yield was calculated as a ratio of the final body weight and carcass weight. The abdominal fat, breast and thigh muscles without bones, wings and hulls were weighed. Their respective percentage values based on carcass weight were calculated.

Blood samples were collected from the jugular veins of 12 birds (3 for each replicate) on the last day of fattening, using disposable sterile syringes. The blood was then transferred to sterilised centrifuge tubes. To determine the bone mineral profile, 12 pieces of tibia bones (3 from each replicate) from each group were used.

The samples of the breast and thigh meat from 12 broiler chickens were stored at 4 ± 2 °C until meat quality analysis of the following parameters: dry matter, water, fat and protein contents, lipid oxidation via determination of malondialdehyde (MDA) concentration and sensory evaluation. For MDA determination and sensory assessment of breast and thigh meat after seven days of storage, samples were stored at 4 ± 2 °C for seven days, until required for use in analyses. Samples for determination of fatty acid profiles were kept at −20 ± 2 °C for up to one month.

### 2.5. Bone and Blood Variables Determination

Total cholesterol, lipids and triglyceride levels were determined from blood serum using a method described by Tietz [28]. For HDL-cholesterol determination, a method described by Sugiuchi et al. [29] was used. The concentration of LDL-cholesterol was determined by a method described by Bachorik [30]. The amount of HDL-cholesterol was enzymatically determined with cholesterol esterase and cholesterol oxidase. Determination of aspartate aminotransferase (AST), alkaline phosphatase (ALP), alanine transaminase (ALT), calcium and phosphorus were based on the respective absorbance measurements. The concentrations of these enzymes and minerals were determined spectrophotometrically [28]. For all spectrophotometric methods, a Cobas C111 biochemistry analyser (Roche diagnostics Ltd., Basel, Switzerland) was used.

The bone analysis was conducted according to Onyango et al. [31]. Calcium was determined by flame atomic absorption spectroscopy at 422.7 nm (Perkin Elmer Analyst 100, PerkinElmer Inc., Waltham, MA USA). Total phosphorus in the samples was determined by the colorimetric method using ammonium molybdate at 620 nm (Spekord 210 Plus, Analytik Jena AG, Jena, Germany).

### 2.6. Meat Quality Analysis

Content of dry matter was determined by oven-drying at 105 °C [32] using a Universal Oven UN 110 (Memmert GmbH + Co. KG, Büchenbach, Germany). A Kjeltec auto type 1030 analyser (Tecator Co., Hoganas, Sweden) was used to determine the crude protein content. Lipids were isolated in ground samples with petroleum ether in Soxhlet apparatus (LTHS 500, Brnenská Druteva v.d., Brno, Czech Republic) and were determined gravimetrically. Fatty acid composition of breast and thigh meat samples was determined by an evaluation of their methyl ester content via gas chromatography, according to Čertík et al. [24], as described above.

To determine the lipid oxidation changes of breast and thigh muscles, the 2-thiobarbituric acid spectrophotometric method was used. The extent of lipid oxidation involved the measurement of thiobarbituric acid reactive substances (TBARS), as prescribed by the method of Reitznerová et al. [33]. TBARS values were measured spectrophotometrically at 532 nm (Helios *α*, v.4.6 Thermo Spectronic, Cambridge, UK). TBARS values were determined within 24 h after slaughter and after 7-day storage in a refrigerator (+4 °C). Results were quantified as malondialdehyde (MDA) equivalents and expressed as mg of malondialdehyde/kg of sample.

### 2.7. Sensory Evaluation

The breast and thigh meat samples were portioned into square cubes (25 × 25 × 25 mm) using a wire slicer, and each cube weighed approximately 25 g. Meat samples were served in white plastic dishes after being cooked in boiled water (until the temperature of 80 °C was measured in the core of the meat) and coded with three-digit random numbers. Samples were served at a temperature of 20 ± 2 °C. Mineral water was provided for mouth-rinsing. The sensory evaluation was carried out in a standardised sensory laboratory (ISO 8589, 2014) built in the Institute of Postgraduate Education of Veterinary Medicine in Košice (Košice, Slovakia). The sensory evaluation was performed by a panel consisting of staff from the University of Veterinary Medicine and Pharmacy in Košice (Košice, Slovakia). The panel consisted of 10 panelists, aged between 30 and 65. All the assessors were trained in the sensory analysis of chicken meat prior to the analysis. During the training period (three months), assessors attended a number of group sessions in which they tasted broiler chicken meat. Selected descriptors were stated during this period for the purposes of the presented study (juiciness and brittleness). Subsequently, all the assessors evaluated overall aroma, taste, appearance and acceptability of each sample on a nine-point hedonic scale (1: dislike extremely, 2: dislike very much, 3: dislike moderately, 4: dislike slightly, 5: neither like nor dislike, 6: like slightly, 7: like moderately, 8: like very much, 9: like extremely). The juiciness and brittleness of the served breast and thigh meat samples were evaluated using a 10-cm structured line scale.

### 2.8. Statistical Analysis

The results obtained in this experiment were expressed as mean ± standard deviation (SD). Analysis of variance (ANOVA) and Tukey’s test for multiple comparisons of means at a significance level of *p* < 0.05 were carried out via the software GraphPad Prism 8.3 (GraphPad Software, San Diego, CA, USA). The effects of 10% addition of wheat bran after SSF by *Cunninghamella echinulata* ATHUM 4411 in broiler diet and supplementation of wheat bran with agrimony extract (*Agrimonia eupatoria* L.) were set as the main factors. Multiple factor analysis was conducted in R-statistics software [34] with the “FactomineR” [35] and “Factoextra” packages [36] according to Semjon et al. [37]. The multiple factor analysis method created a visual form of the results by two plots: correlation circle and graph of individuals. The correlation circle shows the relationship between the analysed variables, the quality of the representation of variables and the correlation between variables and the two extracted dimensions. Positively correlated variables are visualised on the plot together, whereas negative ones are positioned on opposite sides of the plot. The distance between variable points and the origin measures the quality of the variable on the factor map. The graphs of the individuals show representations of individuals in which individuals that are much closer have similar values for all variables in all the groups [38].

## 3. Results

### 3.1. Feed Sample Analysis

The fatty acid profiles of the feed mixtures are shown in Table 2. The solid-state fermentation process produced FF with 7.10% of GLA and showed a higher proportion of linoleic acid (LA) and *α*-linolenic acid (ALA) when compared to the control feed. On the contrary, the oleic acid content was significantly lower in FF, but ALA and LA levels increased in control feed. Control feed did not contain this fatty acid, but the following values were measured in the feed mixtures of the experimental diets: 0.51 and 0.56% of GLA. This represents 0.26 and 0.23 mg/g of gamma-linolenic acid in feed, respectively.

### 3.2. Bone and Blood Analysis

The results of the selected blood biochemical variables and broiler bone determinations are presented in Table 3.

Between the groups of experimental groups of animals (FF10 and FF10 + AE), a significant difference was determined between the obtained ALP blood variables (*p* < 0.05). The FF10 group of animals had a higher average content of ALP, but in this variable, the highest standard deviation was also observed. Total lipids were significantly affected by the experimental diet (*p* < 0.05). The increase of triglyceride (TG) content after the provision of the experimental diet to groups FF10 and FF10 + AE was significant (*p* < 0.05), but in the HDL blood variable, a significant decrease was observed (*p* < 0.05). The calcium and phosphorus contents were neither affected by the supplementation of 10% FF nor by the added AE. The bone variables showed wide ranges in the measured values (*p* > 0.05).

### 3.3. Broilers Production Parameters

Production variables of broilers (weight, feed consumption, feed increment and conversion) after feeding 10% of the fermented feed and in combination with agrimony extract are presented in Table 4. Differences in production variables among the experimental groups were observed in total feed consumption and average daily intake (*p* < 0.05).

The lowest feed consumption among the experimental groups of animals was observed in the experimental group with the 10% addition of FF (*p* < 0.05). Total feed consumption of the FF10 experimental group decreased, compared to the C group (*p* < 0.05). However, the 10% supplementation of FF with AE increased this production variable significantly (*p* < 0.05). The lowest final weight was recorded in FF10, as well as total weight gain, but the differences among experimental groups of broilers in this production variable were insignificant (*p* > 0.05). On the other hand, the highest final weight and highest weight gain were observed in the FF10 + AE experimental group (Table 4). A similar effect of broilers fed the experimental diet supplemented with SSF and AE was observed on the average daily intake variable. The supplementation of SSF in the FF10 group decreased the daily intake of feed, but the addition of AE showed an increase in the trend of daily intake (*p* < 0.05).

The carcass yield results are shown in Table 5. The higher carcass yields were observed in both experimental groups (FF10 and FF10 + AE) than in the C group after evisceration. However, differences in the variables of the carcass yield after slaughtering the animals were observed only in the wing proportion variable (*p* > 0.05).

### 3.4. Meat Quality Variables

The results of the chemical composition of produced breast meat samples are shown in Table 6. In contrast, the dry matter, and thus the water content, of experimental breast meat samples differ significantly in the FF10 + AE group when compared with C and FF10 (*p* < 0.05) groups. The dry matter, water content and fat of thigh meat samples differ significantly among the experimental groups (*p* < 0.01).

**Figure 1:**
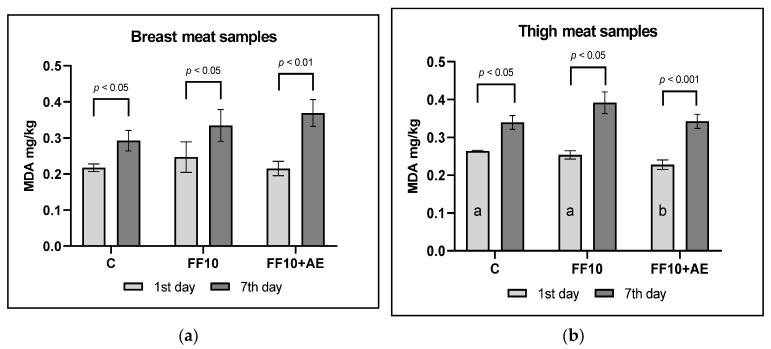
(**a**) The results of malondialdehyde (MDA) content in breast meat samples during storage at 4 ± 2 °C (mg/kg means ± SD). (**b**) The results of malondialdehyde content in thigh meat samples during storage at 4 ± 2 °C (mg/kg means ± SD). C: control group; FF10: broilers fed with a diet enriched with supplementation of 10% FF; FF10 + AE: broilers fed with a diet enriched with 10% supplementation of FF supported with 0.2% agrimony extract ^a, b^ Means in bars with a different superscript letter are statistically different (Tukey’s test, *p* < 0.05).

Lipid oxidation changes in meat samples that were kept in storage at 4 ± 2 °C for seven days are expressed as MDA mg/kg. The results of MDA determination in breast and thigh meat samples during storage are presented in Figure 1. On the first day of storage, differences in MDA content were not observed between each group (*p* > 0.05). The storage of meat samples for seven days at 4 ± 2 °C caused a significant increase in MDA content in all meat samples (Figure 1). However, the effect of supplementing the diet of broilers with SSF or SSF with the addition of AE was observed only in thigh meat samples on the first day of storage (*p* < 0.01).

The results of the breast and thigh meat fatty acid profiles are shown in Table 7. The supplementation of 10% of SSF, as well as a combination of 10% SFF and AE, show a significant impact on the fatty acid profiles of the fat composition of breast meat. The increase in GLA content in breast meat samples for both experimental groups was significant (*p* < 0.001). 

In both experimental groups, FF10 and FF10 + AE, the proportion of oil (OA), linoleic (LA) and dihomo-gamma linolenic (DGLA) fatty acids increased in breast meat (*p* < 0.05). However, the proportion of arachidonic acid (ARA), eicosapentaenic acid (EPA), docosapentaenic acid (DPA) and docosahexaenic acid (DHA) in the breast meat of the experimental groups decreased (*p* < 0.05). The proportion of unsaturated fatty acids, essential fatty acids and n-3 PUFA decreased in both experimental groups (*p* < 0.05) and a higher n-6/n-3 PUFA ratio was also observed than for the C group (*p* < 0.05).

As described in Table 7, the fatty acid profile of thigh meat samples was also affected by the 10% supplementation of the FF and 0.2% addition of AE. A significant increase of ARA and DPA contents in thigh meat samples was observed between control, FF10, and FF10 + AE groups (*p* < 0.05).

**Figure 2:**
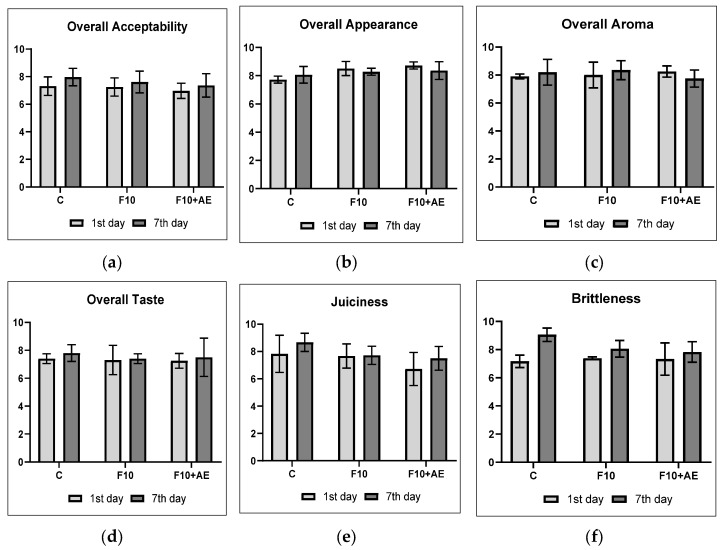
The results of sensory evaluation of breast meat samples (means ± SD): (**a**) overall acceptability, (**b**) overall appearance, (**c**) overall aroma, (**d**) overall taste, (**e**) juiciness and (**f**) brittleness. C: control group; FF10: broilers fed with a diet enriched with supplementation of 10% FF; FF10 + AE: broilers fed with a diet enriched with 10% supplementation of FF supported with 0.2% agrimony extract.

Figure 2 illustrates the results of the sensory evaluation of breast meat samples on the first and seventh days of storage at 4 ± 2 °C. The supplementation with 10% of FF and supporting the FF with the addition of AE in the experimental group did not affect the overall sensory evaluation of breast and thigh meat with respect to appearance, aroma, taste and acceptability of the samples. The juiciness and brittleness of the breast meat samples were also not affected by the supplementation of FF, nor by the addition of AE (*p* > 0.05). Although these differences among the evaluated samples were not statistically significant, an increasing trend in hedonic evaluation and juiciness and brittleness intensities of breast meat samples on the seventh day of storage of breast meat samples were observed.

**Figure 3:**
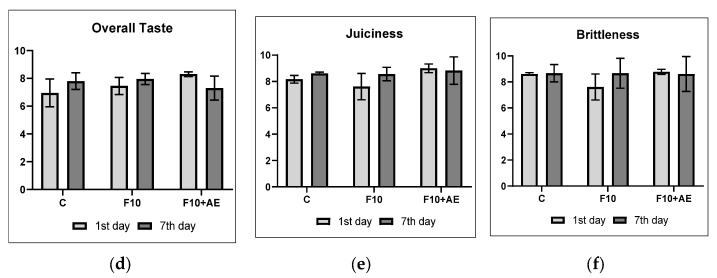
The results of sensory evaluation of thigh meat samples (means ± SD): (**a**) overall acceptability, (**b**) overall appearance, (**c**) overall aroma, (**d**) overall taste, (**e**) juiciness and (**f**) brittleness. C: control group; FF10: broilers fed with a diet enriched with supplementation of 10% FF; FF10 + AE: broilers fed with a diet enriched with 10% supplementation of FF supported with 0.2% agrimony extract.

Identical parameters were examined in thigh meat samples of the experimental groups (C, FF10 and FF10 + AE) and are graphically summarised in Figure 3. The following observations could be made with respect to higher hedonic evaluation and higher intensities of evaluated samples during storage (Figure 3): the points of hedonic evaluation and intensities given by the evaluators of the thigh meat samples did not significantly differ among the experimental groups. The sensory evaluation of breast and thigh meat samples was slightly improved after 7 days of storage. It is beneficial that neither FF nor AE affects the sensory quality of broiler meat because of its fatty acid profile and other physicochemical changes that are mostly related to lipid oxidation.

**Figure 4:**
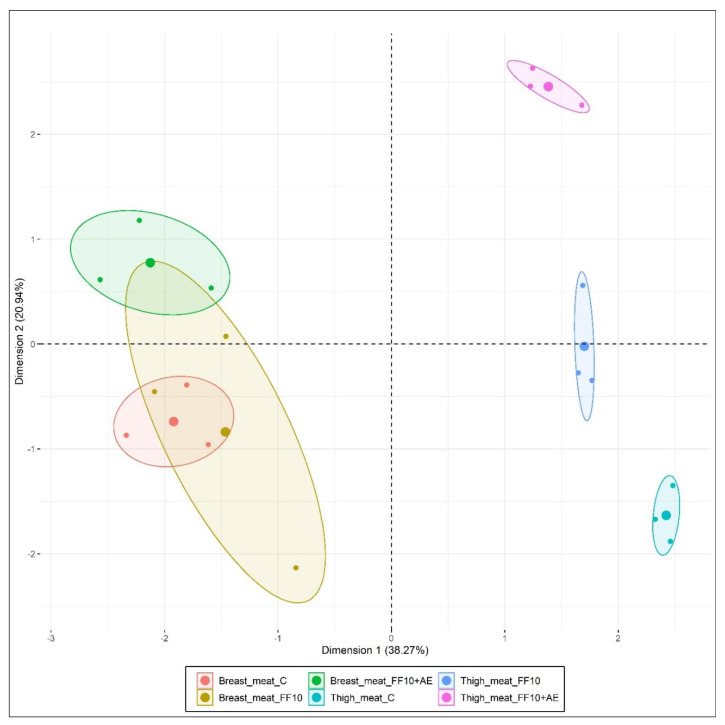
Multiple factor analysis plot of breast and thigh meat samples. C: control group; FF10: broilers fed with a diet enriched with supplementation of 10% FF; FF10 + AE: broilers fed with a diet enriched with 10% supplementation of FF supported with 0.2% agrimony extract.

In this study, multiple factor analysis was applied to the obtained data of the physicochemical results, fatty acid profile analysis, lipid oxidation measurements, and sensory analysis of the breast and thigh meat samples on the first day of storage, whereby meat type and supplementation of the broilers diet with 10% FF and 0.2% addition of AE were identified as the main factors. The analysis extracted the most significant variables, with a minimum loss of information. The results of multiple factor analysis showed four selected components that explain more than 85% of the total variation in the dataset. The first dimension (Dim1) explains 38.27% of variation, Dimension 2 (Dim2) 20.94%, Dimension 3 (Dim3) 14.58%, and Dimension 4 (Dim4) more than 12% (Figure 4).

Contribution of the analysed data in Dim 1 was related to physicochemical variables (25.92%, *r* = 0.97) and the sort of meat samples (26.04%, *r* = 0.97). The highest contribution in Dim1 included fat content (*r* = 0.96), dry matter (*r* = 0.89), water (*r* = −0.89) and protein content (*r* = −0.94) of the breast and thigh meat samples (*r* = 0.94), which correlated at a statistically significant level (*p* < 0.001). Dim2 was characterised by the contribution of sensory variables (21.48%, *r* = 0.73) and the lipid oxidation variable, represented as the determined malondialdehyde content of the meat samples (15.55%, *r* = 0.55). The sensory overall taste (*r* = 0.69), overall aroma (*r* = 0.69) and overall acceptability (*r* = 0.56) of the samples correlated significantly with malondialdehyde content (*r* = −0.55) in Dim2 (*p* < 0.05). 

The first two dimensions explained a total of 59.21% of the variance. The control, FF10 and FF10 + AE experimental groups contributed mainly to characterisation of Dim3, with 63.24% (*r* = 0.87). Fatty acid variables of breast and thigh meat samples contributed mainly to Dim4, with 58.88%, and correlation coefficients for fatty acids were determined as follows: ΣEFA (*r* = 0.72), C20:4 n-6 ARA (*r* = 0.71), C22:5 n-3 DPA (*r* = 0.65), C22:6 n-3 DHA (*r* = 0.63), C20:5 n-3 EPA (*r* = 0.65), C20:3 n-6 DGLA (*r* = 0.62), PUFA n-6 (*r* = 0.58), PUFA n-3 (*r* = 0.57), C18:1 n-9 OA (*r* = −0.59), C18:3 n-3 ALA (*r* = −0.59) and C16:1 n-7 POA (*r* = −0.73) at statistically significant levels (*p* < 0.05).

## 4. Discussion

Microbial oils seem to be a suitable source of important PUFA in broiler nutrition. FF, with the desired content of important GLA, was prepared using SSF in the current experiment. We prepared FF with 7.11% of GLA using lower filamentous fungi *Cunninghamella echinulata* ATHUM 4411. Sun et al. [39] stated that the content of GLA ranges between 5% to 8% during SSF with *Yarowia lipolytica*. Bača et al. [21] stated that SSF feed prepared by *Thamnidium elegans* CCF 1456 in broiler nutrition contained up to 15% of GLA. Čertík et al. [40] reported that the fat-forming *Mucorales* fungi, such as *Thamnidium elegans, Cunninghamella echinulata*, *Cunninghamella elegans* and *Mortierella isabelline*, are most suitable for the production of GLA by SSF fermentation. The most suitable species is *Thamnidium elegans*, but the following species are also efficient: *Cunninghamella echinulata*, *Cunninghamella elegans,* and *Mortierella isabellina*. The produced amount of GLA in fermented products depends on the used fungal strain and the extent to which the conditions were optimal during fermentation, especially temperature [41]. The addition of FF to the basic feed mixture increased GLA in the diet of the experimental groups (Table 2). The content of GLA in feed mixtures administered to both experimental groups was in accordance with our previously published studies [12,21] and ranged about 0.5%. This amount should be sufficient for improvement of production parameters, immunity [10] and also the qualitative parameters of the produced meat [7,12]. In addition to the production of PUFA, the particular filamentous fungi used in our experiment (*Cunninghamella echinulata* ATHUM 4411) causes the elimination of antinutritional components via the fermentation process. These fermented agro-industrial wastes are rich and easily digestible sources of usable energy, protein, trace elements, vitamins and antioxidants. For this reason, fermented products may find applications in animal production [42,43].

In our study, 10% of FF supplementation significantly decreased total feed consumption. This result is consistent with the finding reported by Kovalík et al. [12], where 5% supplementation of the feed fermented by *Cunninghamella elegans* also decreased total feed consumption. Moreover, the final body weight and the feed conversion ratio were higher in the experimental group of chicken. On the contrary, in our experiment, the lowest final body weight was recorded in the FF10 group. 

Searching for suitable additives and their combinations is currently needed to achieve an increase of production parameters and to enhance production parameters. AE at 0.2% concentration was administered in drinking water to the experimental group FF10 + AE. This combination resulted in the highest final body weight, even though the feed consumption was comparable with (by being lower than) the control. However, the obtained feed consumption values for both C and FF10 + AE were higher than for group FF10 (Table 4). We suppose that the mechanisms of agrimony extract action could be related to the stimulation of endogenous enzymes, regulation of intestinal microflora and chemical effects. This led to a reduction of pH in the digestive system and improved animal feed intake [44]. Several studies have confirmed that the broilers had a higher final weight after plant extracts addition to feed, but this was usually accompanied by higher feed consumption [45,46]. Our results correspond with referenced works because broilers of the FF10 + AE group reached the highest final body weight, even though the feed consumption was higher in the experimental group without AE addition (Table 4).

Supplementation of FF at 10% concentration and addition of AE at 0.2% in experimental feed mixtures resulted in several changes to some of the following blood biochemical variables: ALP, TG, TL, HDL and Ca (*p* < 0.05). The proportion of triacylglycerol in the blood serum of broilers increased (*p* < 0.05). The results showed that the addition of AE in the diet of broilers could cause a significant decrease in ALP. The HDL cholesterol was lower in the FF10 group than in the C group (*p* < 0.05). In our study, total lipids in blood samples were affected by the experimental diet of broilers. Ide et al. [47] observed the effects of safflower oil rich in linoleic acid, palm oil rich in saturated acids, and oil of evening primrose origin, containing 43% of GLA, on biochemical parameters in blood serum of rats. The authors noted that oil with GLA content contributed to a significant decrease in serum concentration of triacylglycerols, cholesterol and phospholipids in comparison to palm or safflower oil. The concentrations of LDL fractions of cholesterol in rats given GLA oil were also less than one half of those in animals fed palm or safflower oils. These results confirmed that GLA should affect total cholesterol and LDL-cholesterol by decreasing its values. These ingredients could lead to a higher concentration of triacylglycerols in blood serum as well as an increase in fat storage in the body cavity.

It has been confirmed that feed fibre (cellulose, oat flakes) has a positive impact on fat metabolism and, subsequently, the level of lipid metabolites in chicken blood serum [48]. The same point was noted in the study of Boguslawska-Tryk et al. [49]. They claimed that the addition of nonfermentable fibre to feed (lignocellulose in the amount of 0.5–1%) has an antilipidemic effect. The authors observed that a reduction of the TG concentration and a decrease of total cholesterol content resulted in an increase in HDL and a significant decrease in LDL concentrations in broiler serum. On the other hand, Najafi and Torki [50] did not find any reaction to the concentration of total cholesterol, triglycerides and HDL-cholesterol in the blood serum of the broilers after the feeding of fermented products. Agrimony extract did not change LDL cholesterol. Our observations are in accordance with the study conducted by Fébel et al. [51], which indicated that a change in LDL and total cholesterol concentrations occurred due to increased PUFA intake using vegetable oils. An et al. [52] reported that n-3 and n-6 fatty acids differed in their impact on the triglyceride concentration in blood serum, whereby n-6 fatty acids increased levels of TG. High intake of feed enriched with a higher amount of PUFA may reduce TG in the blood serum of broilers by the modulation of genes involved in lipid metabolism [53]. In our experiment, the TG blood variable was found to be significantly higher for both experimental groups (*p* < 0.05). Calder [54] reported that an increase in dietary n-3 fatty acids in broiler diets led to a reduction in plasma TG and cholesterol. This reduction may be related to the role of n-3 fatty acid in the suppression of TG and apolipoprotein synthesis. In birds, TGs are distributed to tissues with blood. This may be caused by using FF. The experimental feeds may not have had higher starch content, but fermentation increases the content of amylases. Amylases help digest starch to monosaccharide glucose, which is stored as TGs in fat tissue. In contrast to mammals, lipogenesis in birds occurs in the liver rather than in adipose tissue. Therefore, fat deposition is dependent on the availability of plasma lipoproteins, which originate from either diet or the liver [55].

The composition of the fatty acid profile of feed used in poultry nutrition significantly affects the fatty acid composition of the meat fat [56,57]. Indirectly, through the modification of animal rations, the modification of the fatty acid composition of food (meat, eggs) is possible. Most of the published work is focused on adjusting the n-3:n-6 PUFA ratio, especially by increasing the proportion of alpha-linolenic acid [58]. Many experiments have shown a close correlation between the amount and profile of fatty acids in feed and in the lipid-fraction of poultry meat [59,60]. Poultry meat enriched by significant long-chain PUFA (GLA, DGLA, and EPA) is an effective way of increasing the intake of these health-beneficial fatty acids in humans. Jaskiewicz et al. [56] indicated that the proportion of ALA in breast muscle fat after feeding *Camelina sativa*, soybean and rapeseed oils increases with the extended period of feeding and with increased doses of fatty acids in the feed. Based on our results, we can conclude that the fatty acid composition of feed influences the fatty acid composition of meat. An increase of GLA was observed in the breast muscle after feeding the fermented feed. The proportion of other significant fatty acids varied depending on feed. Similarly, other authors (Narciso-Gaytán et al. [61], Tres et al. [62] and Cherif et al. [63]) have reported that the fatty acid composition of chicken meat reflected the fatty acid composition of the dietary oils. In the breast meat obtained from members of both experimental groups, fatty acids were represented mostly by palmitic acid, oleic acid and LA, and this composition was related to the fatty acid composition of the conventional feed components, which formed the basis of the feed of both groups.

The fat and total protein content of the breast meat samples obtained from broilers belonging to both experimental groups were neither affected by the 10% FF supplementation nor the addition of AE (*p* < 0.05). We observed an increase in the proportion of palmitoleic acid (POA), OA, LA and DGLA in breast meat samples of FF10, compared to C (*p* < 0.05). The higher content of GLA in breast meat samples was observed in the FF10 group when compared to C. On the other hand, decreases in the following were observed: SA, ARA, EPA, DPA and DHA. The most important examination in the fatty acid profile of thigh meat samples was the difference in the content of GLA between the C group and the two experimental groups (FF10 and FF10 + AE) (*p* < 0.05). In thigh meat samples of both experimental groups (FF10 and FF10 + AE), the share of the following significant PUFA also decreased: ARA, EPA, DPA and DHA (*p* < 0.05). In terms of the produced fat composition, the addition of agrimony extract produced a significant effect. Higher proportions of POA and OA were recorded in breast meat samples. Administration of 0.2% of AE caused an increase in saturated fatty acids and a decrease in unsaturated fatty acids in breast meat samples (*p* < 0.05). Similar results in breast meat fatty acid composition after feeding the essential oils of cinnamon (0.3 g·kg^−1^), clove (0.6 g·kg^−1^) and caraway (0.5 g·kg^−1^) were recorded by Chowdury et al. [64]. Ahmed et al. [65] reported that PUFA values (especially DHA and EPA) increased after addition of pomegranate extract (*Pinica granatum* L.) at doses of 0.5%, 1.0% and 2.0%. They observed a decrease in levels of saturated fatty acids in breast fat. The effect of individual extracts, particularly the aforementioned essential oils of plants, depends on the chemical composition of individual extracts [66]. However, GLAs, as well as other PUFAs, are very prone to oxidation. Several authors have pointed out that after feeding with PUFA-enriched feed sources like fish oil, seaweed, and flaxseed, higher concentrations of PUFA in meat fat was recorded. Subsequently, oxidation damage and lower oxidative stability were observed during storage [67,68]. The results of our experiment show that an increased proportion of monounsaturated and PUFA in broiler meat also increases fat oxidation during sample storage [20,60,66]. Kovalík et al. [12] and Bača et al. [21] stated that the provision of fermented feed at doses of 3% and 5% decrease the oxidative stability of the fats in the produced meat. However, the oxidative changes of fats can be reduced by the addition of antioxidants in the feed. Nkukwana et al. [69] observed that the meat of chickens fed with the oil extracted from the leaves of *Moringa oleifera* showed better oxidative stability than the meat of the control group. Similarly, beta-carotene, produced during fermentation and thus present in the fermented feed product, is an effective antioxidant. The susceptibility of meat to lipid oxidation increases over storage time—this corresponds with the information conveyed from the work presented by Narciso-Gaytán et al. [61]. Their results also indicated that lipid oxidation increases with the proportion of PUFA. The amount of MDA increased in breast muscle from both experimental groups after 7-day storage at 4 ± 2 °C (*p* < 0.05). The meat of broilers fed a diet supplemented with FF was more susceptible to oxidation compared to the meat of broilers from the control group (Figure 1). Improvement in the meat’s oxidative stability and antioxidative effect was observed. *Agrimonia eupatoria* L. has a high antioxidant capacity, probably due to its rich content of coumarins, flavonoids, tannins and terpenoids [70]. The addition of agrimony extract to poultry nutrition can protect fatty acids in meat against oxidation. It is possible to provide the extract through a water source with high efficiency [71]. A significantly higher increase in MDA concentration was observed in the meat of the experimental group due to a higher amount of PUFA in those muscles. Even though meat oxidation stability was lower, the amount of MDA was not high enough to negatively affect the quality of meat. There is no international legislative limit of MDA concentration in meat, but MDA over 0.5 mg/kg indicates some oxidation, and values above 1.0 mg/kg are unacceptable levels according to several studies [72]. The addition of agrimony extract to water provided to chickens also had a significantly positive impact on the oxidative stability of meat. Undeland [73] confirmed the antioxidant potential of plant extracts. In our experiment, the antioxidant effect of agrimony caused less fat damage in the FF10 + AE group.

**Figure 5:**
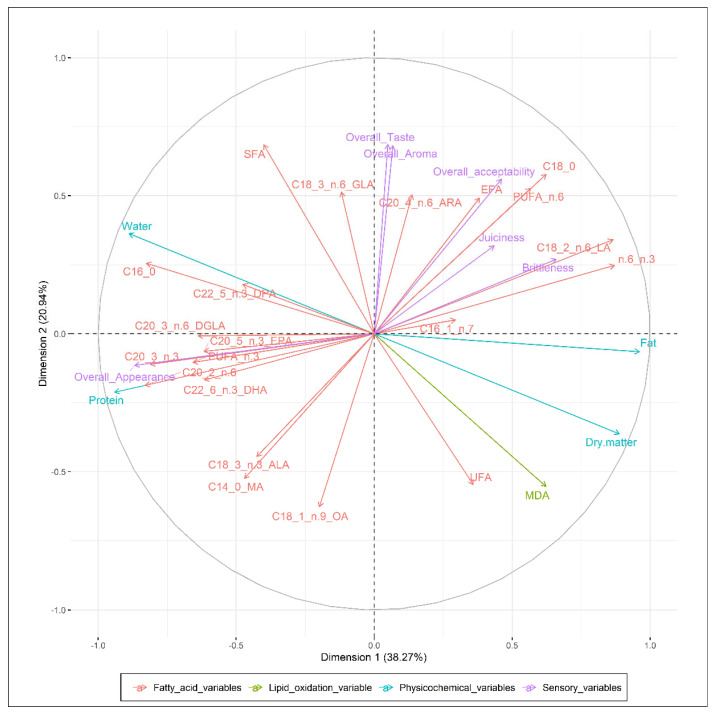
Quantitative variables of multiple factor analysis used for evaluation of breast and thigh meat samples. C: control group; FF10: broilers fed with a diet enriched with supplementation of 10% FF; FF10 + AE: broilers fed with a diet enriched with 10% supplementation of FF supported with 0.2% agrimony extract; SFA: saturated fatty acids; GLA: gamma-linolenic acid; ARA: arachidonic acid; EFA: essential fatty acids; PUFA: polyunsaturated fatty acids; LA: linolenic acid; UFA: unsaturated fatty acids; MDA: malondialdehyde; OA: oleic acid; MA: myristic acid; ALA: alfa-linolenic acid; DHA: docosahexaenic acid; EPA: eicosapentaenic acid; DGLA: dihomo-gamma linolenic; DPA: docosapentaenic acid.

The main advantage of using the multiple factor analysis method in this study is the indication of all examined variables in breast and thigh meat samples, which distinguish the meat quality of nutritionally-enriched broiler meat (Figure 5). Overall, the presented statistical method characterised variables that positively or negatively correlated with each other. According to the applied Kaiser’s criterion [74], the method extracted four selected dimensions, while the first two dimensions explain more than 59% of the total variation in the dataset. The similarity of breast meat samples in the first two dimensions in the analysed parameters was observed between groups FF10 and C and between FF10 and FF10 + AE groups. Breast samples from C and FF10 + AE were different from each other. These groups did not plot close together. On the other hand, the groups of thigh meat samples showed high dissimilarity (Figure 4). The multiple factor analysis method was a very useful and effective statistical tool for the physicochemical and sensory assessments of breast and thigh meat samples of broilers fed a diet supplemented with 10% FF and 0.2% addition of AE as well.

## 5. Conclusions

Based on our results, we can conclude that the supplementation of 10% FF and the addition of agrimony extract to broiler chickens’ diet positively affected the performance parameters, biochemical variables, gamma-linolenic content in fat, and the quality of produced meat. Our results confirm the great potential of solid-state fermentation in production of fermented feed enriched with important fatty acids for the nutrition of broiler chickens. The results also show that the combination of fermented feed with agrimony extract improves the quality of meat and health parameters of chickens. Experiments focused on other meat quality parameters and effects on health status require further investigation on our behalf.

## Figures and Tables

**Table 1:** The composition of broiler diets.

Ingredients	Starter	Grower	Grower + FF	Finisher	Finisher + FF
Corn grain, %	35.00	43.00	41.50	44.00	42.50
Wheat grain, %	26.00	23.00	17.00	26.50	20.00
Soybean meal, %	27.00	22.00	20.70	18.00	17.00
Dehulled sunflower meal, %	2.00	2.50	1.10	2.50	1.10
Rapeseed cake, %	2.00	3.70	3.50	3.20	3.20
Fish meal, %	1.50				
Lard, %	2.50	2.00	3.00	2.40	3.40
Limestone, %	1.55	1.10	1.40	1.05	1.40
Monocalcium Phosphate, %	1.25	1.65	0.75	1.60	0.65
Salt, %	0.20	0.25	0.25	0.25	0.25
Premix ^1^, %	1.00	0.80	0.80	0.50	0.50
Fermented feed, %			10.00		10.00
Dry matter, g/kg	1000.00	1000.00	1000.00	1000.00	1000.00
Crude protein, g/kg	254.61	222.16	222.17	209.14	208.36
Crude fat, g/kg	56.24	55.17	66.43	60.09	71.98
Crude fibre, g/kg	38.20	37.98	43.43	36.62	44.46
Starch, g/kg	454.38	497.13	462.26	504.23	467.74
Ca, g/kg	11.43	9.84	9.60	9.05	8.91
P, g/kg	6.52	5.89	5.81	5.59	5.35
Metabolisable energy, MJ/kg	14.16	14.38	14.18	14.49	14.26

FF: fermented feed; Starter: diet during the first 10 days of fattening; Grower: growing diet from days 11 to 28; Finisher: final diet from days 29 to 35. ^1^ Premix of amino acids, vitamins, trace elements (per kg): lysine 140 g, DL-methionine 180 g, vit. A 1,200,000 IU, D3 500,000 IU, E 2000 mg, pantothenic acid 1800 mg, niacin 6000 mg, choline 60 mg, B6 500 mg, B12 1.8 mg, folic acid 200 mg, copper 1100 mg, iron 8400 mg, zinc 8000 mg, manganese 12,000 mg, iodine 110 mg, selenium 40 mg; Ca: calcium; P: phosphorus.

**Table 2:** Fatty acid profile of feed mixtures.

Ingredients	FF	Grower	Grower + FF	Finisher	Finisher + FF	*p*-Value
C 14:0; MA	0.47 ± 0.06 ^d^	17.56 ± 0.61 ^bc^	16.87 ± 0.01 ^c^	20.15 ± 0.43 ^a^	18.46 ± 0.22 ^b^	<0.001
C 16:0; PA	16.59 ± 0.19 ^c^	21.27 ± 0.14 ^a^	19.94 ± 0.29 ^b^	21.65 ± 0.06 ^a^	20.09 ± 0.07 ^b^	<0.001
C 16:1 n-7; POA	0.55 ± 0.05 ^a^	0.45 ± 0.01 ^bc^	0.51 ± 0.01 ^ab^	0.40 ± 0.02 ^c^	0.46 ± 0.01 ^bc^	0.001
C 18:0; SA	4.34 ± 0.44 ^c^	5.63 ± 0.01 ^a^	5.10 ± 0.01 ^b^	4.59 ± 0.02 ^bc^	4.47 ± 0.01 ^c^	<0.001
C 18:1 n-9; OA	22.72 ± 0.30 ^e^	26.96 ± 0.13 ^a^	26.35 ± 0.18 ^b^	24.09 ± 0.11 ^c^	24.60 ± 0.20 ^d^	<0.001
C 18:1 n-7; VA	1.16 ± 0.04 ^a^	1.17 ± 0.02 ^a^	1.03 ± 0.03 ^b^	0.68 ± 0.02 ^c^	0.72 ± 0.01 ^c^	<0.001
C 18:2 n-6; LA	40.05 ± 1.92 ^a^	24.69 ± 0.05 ^c^	26.95 ± 0.48 ^bc^	27.21 ± 0.48 ^b^	29.26 ± 0.40 ^b^	<0.001
C 18:3 n-6; GLA	7.11 ± 0.80 ^a^		0.51 ± 0.02 ^b^		0.56 ± 0.02 ^b^	<0.001
C 18:3 n-3; ALA	2.35 ± 0.21 ^a^	1.84 ± 0.07 ^bc^	2.08 ± 0.02 ^ab^	1.62 ± 0.02 ^c^	1.84 ± 0.03 ^bc^	<0.001
C 20:0; AA	0.37 ± 0.06	0.36 ± 0.05	0.36 ± 0.02	0.35 ± 0.05	0.35 ± 0.01	0.977
C 20:1 n-9	0.90 ± 0.03 ^a^	0.28 ± 0.03 ^b^	0.30 ± 0.34 ^b^	0.28 ± 0.03 ^b^	0.31 ± 0.03 ^b^	<0.001
C 22:0	0.66 ± 0.01 ^a^	0.25 ± 0.01 ^c^	0.34 ± 0.01 ^b^	0.24 ± 0.01 ^c^	0.35 ± 0.02 ^b^	<0.001
C 24:0	1.31 ± 0.12 ^a^	0.17 ± 0.02 ^c^	0.36 ± 0.04 ^b^	0.18 ± 0.02 ^c^	0.36 ± 0.02 ^b^	<0.001
GLA, mg/g FF	2.15 ± 0.27 ^a^		0.26 ± 0.01 ^b^		0.23 ± 0.01 ^b^	<0.001

FF: fermented feed; Grower: growing diet from days 11 to 28; Finisher: final diet from days 29 to 35; MA: myristic acid; PA: palmitic acid; POA: palmitoleic acid; OA: oleic acid; VA: vaccenic acid; LA: linolenic acid; GLA: gamma-linolenic acid; ALA: alfa-linolenic acid; AA: arachidonic acid. ^a, b, c, d, e^ Means in a row with a different superscript letter are statistically different (Tukey’s, *p* < 0.05).

**Table 3:** The results of blood and bone analysis (means ± SD).

Samples	Variables	C	FF10	FF10 + AE	*p*-Value
Blood	AST, μkat/L	4.66 ± 0.88	4.68 ± 0.02	5.33 ± 0.92	0.488
	ALT, μkat/L	0.05 ± 0.02	0.07 ± 0.01	0.06 ± 0.00	0.419
	ALP, μkat/L	66.00 ± 3.18 ^a, b^	86.56 ± 23.17 ^a^	47.89 ± 7.68 ^b^	0.043
	GMT, μkat/L	0.38 ± 0.07	0.28 ± 0.04	0.30 ± 0.03	0.099
	TCHOL, mmol/L	2.82 ± 0.20	2.68 ± 0.19	2.81 ± 0.29	0.729
	TG, mmol/L	0.37 ± 0.03 ^b^	1.08 ± 0.03 ^a^	0.92 ± 0.11 ^a^	<0.001
	TL, g/L	4.54 ± 0.05 ^b^	5.09 ± 0.30 ^a, b^	5.40 ± 0.71 ^a^	0.134
	HDL, mmol/L	1.00 ± 0.04 ^a^	0.90 ± 0.02 ^b^	0.92 ± 0.04 ^ab^	0.027
	LDL, mmol/L	0.59 ± 0.01	0.52 ± 0.06	0.59 ± 0.09	0.352
	Ca, mmol/L	3.36 ± 0.27 ^a^	2.43 ± 0.00 ^b^	2.51 ± 0.08^b^	0.001
	P, mmol/L	2.53 ± 0.40	2.21 ± 0.07	2.14 ± 0.02	0.178
	Mg, mmol/L	0.86 ± 0.00	0.87 ± 0.02	0.88 ± 0.04	0.714
Bone	Ca, g/kg	226.21 ± 4.58	277.01 ± 95.74	186.32 ±14.66	0.219
	P, g/kg	15.26 ± 0.91	16.58 ± 3.09	16.25 ± 2.15	0.765

C: control group; FF10: broilers fed with a diet enriched with supplementation of 10% FF; FF10 + AE: broilers fed with a diet enriched with 10% supplementation of FF supported with 0.2% agrimony extract; AST: aspartate aminotransferase; ALT: alanine aminotransferase; ALP: alkaline phosphatase; GMT: gamma glutamyltransferase; TCHOL: total cholesterol; TG: triglyceride; TL: total lipids; HDL: high-density lipoprotein; LDL: low-density lipoprotein; Ca: calcium; P: phosphorus; Mg: magnesium; ^a, b^ Means in a row with a different superscript letter are statistically different (Tukey’s test, *p* < 0.05).

**Table 4:** The results of broiler production parameters (means ± SD).

Variables	C	FF10	FF10 + AE	*p*-Value
Body weight 1st day, g	50.00 ± 3.00	50.00 ± 3.00	50.00 ± 3.00	
Body weight 36th day, g	2535.52 ± 68.00	2529.36 ± 72.00	2551.48 ± 52.00	0.904
Total feed consumption, g	3968.13 ± 25.00 ^a^	3884.10 ± 22.00 ^b^	3951.23 ± 30.00 ^a^	0.016
Body weight gain, g	2482.62 ± 64.00	2476.75 ± 68.00	2498.48 ± 48.00	0.912
Feed conversion ratio, g/g	1.60 ± 0.02	1.57 ± 0.02	1.58 ± 0.03	0.355

C: control group; FF10: broilers fed with a diet enriched with supplementation of 10% FF; FF10 + AE: broilers fed with a diet enriched with 10% supplementation of FF supported with 0.2% agrimony extract. ^a, b^ Means in a row with a different superscript letter are statistically different (Tukey’s test, *p* < 0.05).

**Table 5:** The results of the carcass yield of broilers (means ± SD).

Samples	C	FF10	FF10 + AE	*p*-Value
Carcass yield, %	70.80 ± 1.20	71.80 ± 2.10	72.20 ± 1.70	0.611
Breast without bone, %	26.40 ± 0.70	25.60 ± 1.01	25.80 ± 0.96	0.559
Thighs with bone, %	27.70 ± 0.70	26.40 ± 1.50	26.20 ± 1.27	0.323
Wings, %	10.10 ± 0.20 ^a^	8.90 ± 0.20 ^b^	9.40 ± 0.20 ^b^	0.001
Hull, %	32.70 ± 1.70	30.40 ± 1.10	30.60 ± 1.20	0.151
Abdominal fat, %	1.70 ± 0.40	1.60 ± 0.30	1.70 ± 0.40	0.930

C: control group; FF10: broilers fed with a diet enriched with supplementation of 10% FF; FF10 + AE: broilers fed with a diet enriched with 10% supplementation of FF supported with 0.2% agrimony extract. ^a, b^ Means in a row with a different superscript letter are statistically different (Tukey’s test, *p* < 0.05).

**Table 6:** The results of the chemical composition determination of breast meat samples (means ± SD).

Meat Samples	Variable	C	FF10	FF10 + AE	*p*-Value
Breast meat	Dry matter, %	25.05 ± 0.30 ^a^	25.46 ± 0.51 ^a^	24.54 ± 0.15 ^b^	0.051
	Water, %	74.95 ± 0.30 ^a, b^	74.54 ± 0.51 ^b^	75.46 ± 0.15 ^a^	0.051
	Fat, %	2.64 ± 0.17	2.38 ± 0.17	2.43 ± 0.24	0.296
	Total protein, %	21.20 ± 0.20	21.50 ± 0.30	21.10 ± 0.20	0.182
Thigh meat	Dry matter, %	28.88 ± 0.47 ^a^	27.24 ± 0.31 ^b^	26.09 ± 0.27 ^c^	<0.001
	Water, %	71.12 ± 0.47 ^c^	72.76 ± 0.31 ^b^	73.91 ± 0.27 ^a^	<0.001
	Fat, %	10.23 ± 0.43 ^a^	7.68 ± 0.33 ^b^	7.04± 0.37 ^b^	<0.001
	Total protein, %	19.16 ± 0.19	19.38 ± 0.22	19.02 ± 0.23	0.197

C: control group; FF10: broilers fed with a diet enriched with supplementation of 10% FF; FF10 + AE: broilers fed with a diet enriched with 10% supplementation of FF supported with 0.2% agrimony extract. ^a, b^ Means in a row with a different superscript letter are statistically different (Tukey’s test, *p* < 0.05).

**Table 7:** Fatty acid profile of breast and thigh meat samples (means ± SD).

Meat Samples	Variables	C	FF10	FF10 + AE	*p*-Value
Breast meat samples	C14:0; MA, %	1.92 ± 0.04	1.89 ± 0.06	2.04 ± 0.08	0.056
	C16:0; PA, %	20.76 ± 0.18	21.52 ± 0.13	22.01 ± 0.11	<0.001
	C16:1 n-7; POA, %	2.64 ± 0.07 ^c^	3.39± 0.07 ^b^	4.23 ± 0.25 ^a^	<0.001
	C18:0; SA, %	10.99 ± 0.12 ^a^	9.82 ± 0.12 ^b^	10.16 ± 0.45 ^b^	0.005
	C18:1 n-9; OA, %	24.80 ± 0.11 ^c^	25.77 ± 0.14 ^b^	26.54 ± 0.45 ^a^	0.001
	C18:2 n-6; LA, %	17.74 ± 0.15 ^b^	18.67 ± 0.16 ^a^	18.48 ± 0.16 ^a^	0.001
	C18:3 n-6; GLA, %	0.07± 0.01 ^c^	0.17 ± 0.01 ^a^	0.14 ± 0.01 ^b^	<0.001
	C18:3 n-3; ALA, %	0.69 ± 0.01 ^b^	0.68 ± 0.02 ^b^	0.77 ± 0.04 ^a^	0.014
	C20:2 n-6; %	0.89 ± 0.02 ^b^	0.89 ± 0.05 ^b^	0.57 ± 0.04 ^a^	<0.001
	C20:3 n-3; %	0.10 ± 0.01 ^b^	0.12 ± 0.01 ^b^	0.07 ± 0.01 ^a^	<0.001
	C20:3 n-6; DGLA, %	1.62 ± 0.02 ^b^	2.03 ± 0.04 ^a^	1.12 ± 0.05 ^c^	<0.001
	C20:4 n-6; ARA, %	7.17 ± 0.04 ^a^	5.46 ± 0.15 ^b^	4.66 ± 0.30 ^c^	<0.001
	C20:5 n-3; EPA, %	0.70 ± 0.02 ^a^	0.42 ± 0.01 ^b^	0.34 ± 0.02 ^c^	<0.001
	C22:5 n-3; DPA, %	1.36 ± 0.03 ^a^	0.94 ± 0.02 ^b^	0.87 ± 0.05 ^b^	<0.001
	C22:6 n-3; DHA, %	1.27 ± 0.01 ^a^	0.87 ± 0.02 ^b^	0.67 ± 0.04 ^c^	<0.001
	∑ SFA, %	34.45 ± 0.21 ^b^	35.54 ± 0.22 ^b^	36.92 ± 0.25 ^a^	<0.001
	∑ UFA, %	65.55 ± 0.21 ^a^	64.46 ± 0.22 ^a^	63.08 ± 0.25 ^b^	<0.001
	∑ PUFA n-3, %	4.16 ± 0.03 ^a^	3.07 ± 0.02 ^b^	2.77 ± 0.07 ^c^	<0.001
	∑ PUFA n-6, %	26.61 ± 0.17 ^a^	26.34 ± 0.30 ^a^	24.39 ± 0.48 ^b^	<0.001
	n-6/n-3, %	6.40 ± 0.06 ^c^	8.57 ± 0.06 ^b^	8.79 ± 0.09 ^a^	<0.001
	∑ EFA, %	30.76 ± 0.17 ^a^	29.41 ± 0.32 ^b^	27.17 ± 0.54 ^c^	<0.001
Thigh meat samples	C14:0; MA, %	1.99 ± 0.19 ^a^	1.64 ± 0.11 ^ab^	1.46 ± 0.13 ^b^	0.012
	C16:0; PA, %	19.77 ± 0.25 ^a^	20.45 ± 0.42 ^a^	20.51 ± 0.14 ^a^	0.040
	C16:1 n-7; POA, %	4.09 ± 0.20 ^a^	3.81 ± 0.19 ^ab^	3.38 ± 0.39 ^b^	0.052
	C18:0; SA, %	1.01 ± 0.25 ^b^	11.87 ± 0.46 ^ab^	12.74 ± 0.39 ^a^	0.004
	C18:1 n-9; OA, %	27.63 ± 0.06 ^a^	24.44 ± 0.37 ^b^	22.44 ± 0.66 ^b^	<0.001
	C18:2 n-6; LA, %	20.35 ± 0.23 ^b^	21.69 ± 0.46 ^a^	21.19 ± 0.51 ^ab^	0.021
	C18:3 n-6; GLA, %	0.08 ± 0.00 ^b^	0.13 ± 0.02 ^a^	0.15 ± 0.01 ^a^	0.003
	C18:3 n-3; ALA, %	0.74 ± 0.01 ^a^	0.62 ± 0.02 ^b^	0.57 ± 0.04 ^b^	0.001
	C20:2 n-6; %	0.34 ± 0.05 ^b^	0.39 ± 0.01 ^ab^	0.45 ± 0.05 ^a^	0.042
	C20:3 n-3; %	0.04 ± 0.01 ^b^	0.05 ± 0.00 ^ab^	0.06 ± 0.01 ^a^	0.021
	C20:3 n-6; DGLA, %	0.73 ± 0.17 ^b^	1.12 ± 0.01 ^a^	1.29 ± 0.16 ^a^	0.006
	C20:4-n-6; ARA, %	4.95 ± 0.18 ^c^	6.36 ± 0.22 ^b^	7.78 ± 0.19 ^a^	<0.001
	C20:5 n-3; EPA, %	0.23 ± 0.00 ^b^	0.36 ± 0.02 ^a^	0.36 ± 0.02 ^a^	<0.001
	C22:5 n-3; DPA, %	0.73 ± 0.01 ^c^	0.92 ± 0.04 ^b^	1.05 ± 0.06 ^a^	<0.001
	C22:6 n-3; DHA, %	0.57 ± 0.02 ^b^	0.69 ± 0.04 ^a^	0.70 ± 0.06 ^a^	0.022
	∑ SFA, %	34.97 ± 0.58 ^b^	35.85 ± 0.27 ^ab^	36.09 ± 0.13 ^a^	0.024
	∑ UFA, %	65.03 ± 0.58 ^a^	64.15 ± 0.27 ^ab^	63.91 ± 0.13 ^a^	0.024
	∑ PUFA n-3, %	2.36 ± 0.04 ^b^	2.69 ± 0.08 ^a^	2.78 ± 0.12 ^a^	0.002
	∑ PUFA n-6, %	26.11 ± 0.54 ^b^	29.30 ± 0.70 ^a^	30.41 ± 0.80 ^a^	0.001
	n-6/n-3, %	11.08 ± 0.20 ^a^	10.91 ± 0.09 ^a^	10.94 ± 0.20 ^a^	0.461
	∑ EFA, %	28.47 ± 0.56 ^b^	31.99 ± 0.77 ^a^	33.20 ± 0.92 ^a^	0.001

C: control group; FF10: broilers fed with a diet enriched with supplementation of 10% FF; FF10 + AE: broilers fed with a diet enriched with 10% supplementation of FF supported with 0.2% agrimony extract; MA: myristic acid; PA: palmitic acid; POA: palmitoleic acid; OA: oleic acid; VA: vaccenic acid; LA: linolenic acid; GLA: gamma-linolenic acid; ALA: alfa-linolenic acid; DGLA: dihomo-gamma-linolenic acid; AA: arachidonic acid; EPA: eicosapentaenic acid; DPA: docosapentaenic acid; DHA: docosahexaenic acid; SFA: saturated fatty acids; UFA: unsaturated fatty acids; PUFA: polyunsaturated fatty acids; EFA: essential fatty acids. ^a, b, c^ Means in a row with a different superscript letter are statistically different (Tukey’s test, *p* < 0.05).
